# Cell death-based treatment of glioblastoma

**DOI:** 10.1038/s41419-017-0021-8

**Published:** 2018-01-25

**Authors:** Simone Fulda

**Affiliations:** 1 Institute for Experimental Cancer Research in Pediatrics, Goethe-University, Komturstr. 3a, 60528 Frankfurt, Germany; 2 German Cancer Consortium (DKTK), partner site Frankfurt, Germany; 3 German Cancer Research Center (DKFZ), Heidelberg, Germany

## Abstract

Cancer cells including glioblastoma have typically evolved multiple mechanisms to escape programmed cell death in order to maintain their survival. Defects in cell death mechanisms not only facilitate tumorigenesis but also ensure resistance to current anticancer therapies. This emphasizes that targeting cell death pathways may provide a means to tackle one of the Achilles’ heels of cancer. Over the last decades several approaches have been developed to selectively target cell death pathways for therapeutic purposes. Some of these concepts have already been transferred into clinical application in oncology and may open new perspectives for the treatment of cancer.

## Facts

- Cell death pathways are frequently inactivated in human cancers, including glioblastoma.
- Reactivation of programmed cell death represents a promising therapeutic strategy for glioblastoma.
- There are already prime examples of how targeting cell death pathways can be translated into clinical application.

## Open questions

- What are suitable predictive biomarkers to select patients for clinical trials with cell death-targeting therapeutics?
- Which are the most suitable combination partners for cell death targeting therapeutics?
- How can systems medicine approaches be exploited to predict the capability of cancer cells to undergo cell death?

## Introduction

Programmed cell death represents a cellular process that has to a high degree been conserved during evolution^1^. Apoptosis is one of the most extensively studied forms of programmed cell death. There are several morphological characteristics of cells undergoing apoptosis, for example cell shrinkage, membrane blebbing chromatin condensation and nuclear fragmentation^1^. On the molecular level, two major signaling pathways have been delineated that are involved in the regulation of apoptotic cell death and eventually lead to the activation of caspases as a common effector mechanism of apoptosis. Caspases represent a family of cysteine proteases that are present as inactive proforms in the cytosol and become activated during apoptosis by their proteolytic cleavage at specific aspartate residues^2^. A very large set of targets that are proteolytically processed by caspases have been identified that contribute to the execution of apoptosis or are involved in cellular signaling events^2^.

**Fig. 1:**
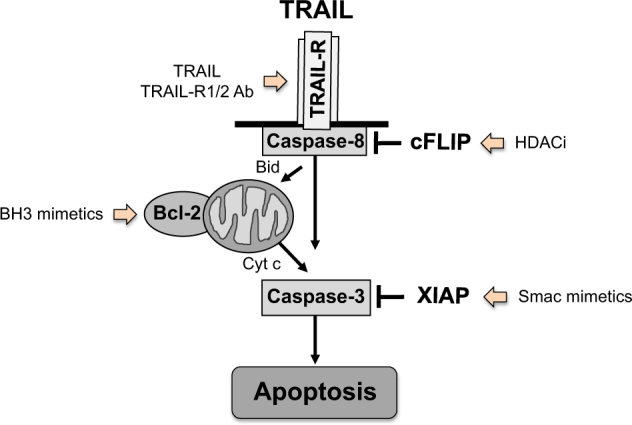
Apoptosis signaling pathways

In the death receptor (extrinsic) pathway (Fig. 1), apoptosis is initiated upon triggering of death receptors on the cell surface by their cognate death receptor ligands. Death receptors belong to the superfamily of tumor necrosis factor (TNF) receptors, including for example TNF-receptor 1 (TNFR1), Fas/APO-1/CD95 and death receptors 4 and 5, also named TRAIL-R1 and TRAIL-R2^3^. The corresponding death receptor ligands, i.e., TNFα, Fas ligand, TNF-related apoptosis-inducing ligand (TRAIL), are either soluble or transmembrane proteins that are exposed, for example, on the plasma membrane of immune cells. Ligation of death receptors initiates their oligomerization and recruitment of adaptor proteins such as FADD (Fas-associated death domain) or TRADD (TNFR type 1-associated death domain) and initiator caspases such as caspase-8, thereby forming the death-inducing signaling complex (DISC). Within the DISC, caspase-8 is auto-proteolytically cleaved and can subsequently activate effector caspases such as caspase-3. In addition, caspase-8-mediated proteolytic processing of BID into tBID favors the translocation of tBID to mitochondrial membranes where it engages the intrinsic pathway of apoptosis (Fig. 1).

In the mitochondrial (intrinsic) pathway (Fig. 1), various upstream cellular stress signals, for example DNA damage, growth factor deprivation or reactive oxygen species (ROS), can trigger the release of mitochondrial proteins such as cytochrome c or Smac from the intermembrane space into the cytosol^4^. Cytochrome c promotes apoptosis by engaging caspase-3 activation (Fig. 1). Smac binds to Inhibitor of Apoptosis (IAP) proteins such x-linked IAP (XIAP), which disrupts the interaction of XIAP with caspase-3, 7, and 9, thereby promoting caspase activation^5^. In type II cells such as glioblastoma cells, the engagement of the mitochondrial pathway is required for full activation of downstream effector mechanisms by amplifying the apoptotic signal^6^.

Besides apoptosis, additional regulated cell death programs have been delineated in recent years, for example necroptosis or autophagic cell death^7^, which however will not be in the focus of the present review.

One of the key hallmarks of cancer is the ability to evade programmed cell death^8^. Cell death programs can be inactivated or be defective due to multiple causes. Evasion of cell death can foster tumor formation and progression^8^. In addition, blockage of cell death is a frequent cause of treatment resistance, since the efficacy of most current anticancer therapies critically depends on intact signal transduction pathways to cell death^9^.

Glioblastoma represents the most frequent primary malignant brain tumor. Current treatment options are limited and include surgery, radiation and chemotherapy with the alkylating agent temozolomide (TMZ)^10^. Despite aggressive therapies, patients with glioblastoma usually have a very poor prognosis^11^. This highlights the high medical need to develop new treatment strategies.

Against this background, reactivation of defective cell death programs is currently considered as a promising approach for the treatment of cancer^9^. This review intends to discuss prototypic strategies aiming at targeting programmed cell death in glioblastoma cells (Fig. 1).

## Targeting IAP proteins

**Fig. 2:**
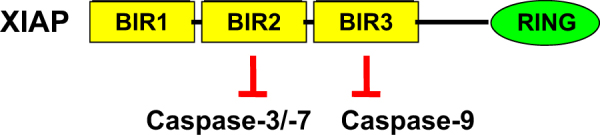
Caspase inhibition by XIAP

IAP proteins are a family of eight antiapoptotic proteins that have been highly conserved throughout evolution^5^. All IAP proteins contain at least one baculovirus IAP repeat domain, which contains 70–80 amino acids that mediate the interaction between IAP proteins and other proteins such as caspases (Fig. 2)^5^. Some IAP proteins such as XIAP, cellular IAP (cIAP)1, and cIAP2, also harbor the Really Interesting New Gene domain (RING) which functions as E3 ubiquitin ligase (Fig. 2)^5^. XIAP blocks apoptosis by binding to and inhibiting caspases such as caspase-3, 7, and 9, while cIAP1 and cIAP2 ensure survival by promoting NF-κB activation^5^.

Smac mimetics mimick the endogenous mitochondrial protein Smac and bind to IAP proteins such as XIAP, cIAP1, and cIAP2^5^. Smac mimetics initiate apoptosis by antagonizing IAP proteins which may engage, via NF-κB activation, an autocrine/paracrine TNFα/TNFR1-dependent signaling loop that has been implicated in mediating Smac mimetic-induced cell death^12,13^. In addition to TNFα, death receptor (DR5) has been identified by genome-wide gene expression analyses as another key mediator of Smac mimetic-induced apoptosis, especially in those cancer cell lines including glioblastoma cells that undergo Smac mimetic-induced apoptosis largely independently of TNFα/TNFR1^14^. This Smac mimetic-stimulated upregulation of DR5 occurred in a NF-κB-dependent manner and was critically required for apoptosis, as knockdown of DR5 significantly inhibited Smac mimetic-induced formation of a Receptor-interacting protein (RIP)1/FADD/caspase-8-containing cytosolic cell death complex, caspase activation and apoptosis^14^. In primary, patient-derived glioblastoma cells, remarkable differences in responses to the Smac mimetic Birinapant, both as single agent and in combination with TMZ, have recently been recorded^15^.

Smac mimetics have also been shown to sensitize glioblastoma cells toward TMZ, the first-line anticancer drug used for treating patients with glioblastoma^16^. This synergy was shown to involve enhanced loss of mitochondrial membrane potential (MMP), release of cytochrome c, activation of caspases and caspase-dependent apoptosis^16^. Also, Smac mimetics acted in concert with TMZ to stimulate the assembly of a multiprotein complex composed of RIP1, caspase-8, and FADD^16^. RNAi-mediated silencing studies confirmed that RIP1 is indeed required for cell death caused by Smac mimetic/TMZ^16^. By comparison, an autocrine/paracrine TNFα/TNFR1 signaling, which has been implicated in mediating the cytotoxicity of Smac mimetics as single agents^12,13^, has been found to be dispensable for Smac mimetic/TMZ-triggered cell death^16^. Interestingly, Smac mimetic-mediated NF-κB stimulation turned out to be necessary, since blocking NF-κB activation rescued glioblastoma cells from apoptosis^16^.

Whole-genome gene expression profiling performed to discover Smac mimetic-stimulated NF-κB target genes that are responsible for the synergistic interaction of Smac mimetics and TMZ resulted in the identification of interferon-β (IFN β) as a critical target gene that was upregulated in an NF-κB-dependent manner^17^. Knockdown of IFNβ or the corresponding receptor IFNα/β receptor (IFNAR) conferred protection from Smac mimetic/TMZ-induced cell death^17^. Also, IFN-mediated proapoptotic signaling involving upregulation of the proapoptotic BCL-2 family proteins BAX and PUMA contributed to the induction of apoptosis^17^.

In addition to triggering IFN signaling, Smac mimetics acted in concert with TMZ to increase the generation of cytosolic as well as mitochondrial ROS, which contributed to activate BAX^18^. Rescue experiments showing that various ROS scavengers protected glioblastoma cells from Smac mimetic/TMZ-induced BAX activation and cell death confirmed the functional relevance of ROS production in this model of apoptosis^18^.

In addition to DNA-damaging drugs, Smac mimetics have been reported to synergistically increase the radiosensitivity of glioblastoma cells^19,20^. Smac mimetic-conferred radiosensitization occurred in an NF-κB-dependent manner, as genetic blockage of NF-κB rescued this synergy^19^. Also, overexpression of full-length or the mature form of Smac has been shown to significantly enhance γ-irradiation-induced apoptosis and to reduce clonogenic survival of glioblastoma cells^21^.

Furthermore, Smac mimetics have been described to cooperate with agents modulating immune cell-mediated death of cancer cells. For example, subtoxic concentrations of Smac mimetics cooperate with IFNα to trigger cell death in a panel of solid tumor cell lines including glioblastoma cells in a highly synergistic manner^22^. Of note, a differential requirement of TRAIL or TNFα as mediators of IFNα/Smac mimetic-induced cell death has been identified depending on the cellular context^22^. Interestingly, inhibition of paracrine/autocrine TNFα signaling has been found to rescue HT-29 colon carcinoma cells, but not A172 glioblastoma cells from IFNα/Smac mimetic-induced cell death^22^. In contrast, blockage of TRAIL signaling through genetic silencing of TRAIL or its cognate receptor DR5 significantly protected A172 glioblastoma cells from IFNα/Smac mimetic-induced cell death^22^. Despite this differential requirement of TRAIL signaling in glioblastoma cells, both TNFα and TRAIL expression was upregulated by IFNα/Smac mimetic cotreatment^22^. Interestingly, A172 cells proved to be resistant to exogenously added recombinant TNFα even in the presence of Smac mimetics, whereas they display a high sensitivity towards TRAIL/Smac mimetics^22^. This demonstrates that a differential sensitivity towards TRAIL or TNFα can determine the dependency on either death receptor ligand for IFNα/Smac mimetic-induced cell death.

Recently, Smac mimetics have been reported to synergize with immune checkpoint inhibitors to enhance tumor immunity in preclinical models of glioblastoma^23^. A combinatorial approach using Smac mimetics together with innate immune stimulants and immune checkpoint inhibitors was shown to result in durable responses in mouse glioblastoma models, which was dependent on cytotoxic T-cell activity, type I IFN, and TNFα signaling^23^.

Besides increasing the sensitivity of glioblastoma cells to cell death, Smac mimetic have been shown to promote migration and invasion of glioblastoma cells in vitro as well as infiltrative tumor growth in vivo^24,25^. These non-apoptotic functions of Smac mimetics have been observed at low, non-toxic concentrations of Smac mimetics and were dependent on non-canonical NF-κB signaling^24,25^. Molecular studies showed that Smac mimetic-stimulated NF-κB activation transcriptionally activated TNFα, thereby increasing expression levels of chemokines/cytokines involved in migration and invasion^25^. Of note, pharmacological or genetic inhibition of NF-κB or TNFα/TNFR1 signaling rescued Smac mimetic-stimulated migration and invasion^25^. Whole-genome expression analyses identified chemokine (C–C motif) ligand 2 (CCL2) as the top-listed NF-κB-regulated gene that was increased in glioblastoma cells by Smac mimetic treatment^24^. This Smac mimetic-stimulated upregulation of CCL2 followed by its secretion into the supernatant has been shown to mediate Smac mimetic-imposed migration and invasion, as CCL2 silencing abrogated these effects^24^. Also paracrine effects on the tumor microenvironment might contribute to this effect, as co-culture experiments revealed that Smac mimetic-treated glioblastoma cells can stimulate the migration of non-malignant astroglial cells toward glioblastoma cells via secretion of CCL2 by glioblastoma cells^24^. These findings indicating that Smac mimetics may cause potentially undesirable therapeutic effects have important implications for the use of Smac mimetics as cancer therapeutics.

Furthermore, Smac mimetics have been shown to promote the differentiation of glioblastoma cancer stem-like cells in an NF-κB-dependent manner^26^. Accordingly, Smac mimetics transcriptionally activated the astrocytic marker GFAP via NF-κB without altering expression of the neuronal marker β-III-tubulin^26^. This astrocytic differentiation of glioblastoma cancer stem-like cells was accompanied by downregulation of stemness markers such as CD133, Nanog and Sox2. In contrast to glioblastoma cancer stem-like cells, Smac mimetics do not alter differentiation nor expression of stemness markers in non-malignant neural stem cells^26^. The Smac mimetic-conferred differentiation of glioblastoma cancer stem-like cells reduced their in vitro and in vivo clonogenicity as well as their tumorigenicity in orthotopic and subcutaneous mouse models, leading to increased survival of mice^26^. Thus, Smac mimetic-induced non-apoptotic functions might have important implications for targeting glioblastoma cancer stem-like cells. Currently, a number of Smac mimetics are being evaluated in early clinical trials either as monotherapy or in combination studies in solid tumors as well as in hematological malignancies^27^.

## Targeting death receptor-induced apoptosis

Another possibility to reactive programmed cell death in cancer cells is to engage death receptors on the cell surface, in particular TRAIL-R1 and TRAIL-R2^3^. As to the relevance of the TRAIL receptor/ligand system in glioblastoma, TRAIL receptors have been documented to be present in primary glioblastoma samples^28,29^. Tumor cell-selective induction of apoptosis by TRAIL has been observed in glioblastoma cells but not in non-transformed astrocytes^30,31^, in line with the notion that TRAIL preferentially triggers apoptosis in tumor versus normal cells^32^. Interestingly, a proapoptotic function of NF-κB has been identified during TRAIL-induced apoptosis in glioblastoma cells, as genetic inhibition of NF-κB by overexpression of a dominant-negative IκBα-superrepressor rescued glioblastoma cells from TRAIL-induced apoptosis^33^. Vice versa, genetic activation of NF-κB by ectopic expression of constitutively active IκB kinase complex (IKK)β significantly enhanced TRAIL-stimulated apoptosis^33^. On the molecular level, NF-κB inhibition attenuated the recruitment of FADD and caspase-8 and formation of the DISC following treatment with TRAIL, leading to reduced caspase activation, loss of MMP and cytochrome c release^33^. In contrast to TRAIL, NF-κB inhibition strongly increased TNFα-stimulated apoptosis^33^. Interestingly, comparative studies revealed that TRAIL triggers apoptosis before transcriptional activation, while TNFα rapidly upregulates antiapoptotic proteins by stimulating their transcription^33^.

Aside from using TRAIL as monotherapy, various combinations of TRAIL together with additional cancer therapeutics have been developed over the years in order to maximize the antitumor activity of TRAIL. For example, recombinant soluble TRAIL or agonistic TRAIL receptor antibodies have been combined with small-molecule inhibitors of IAP proteins such as Smac mimetics. Smac mimetics have been described to synergistically induce apoptosis and to reduce colony formation when applied together with the TRAIL-R2-specific antibody Drozitumab in several glioblastoma cell lines, primary glioblastoma cultures as well as glioblastoma stem-like cells^34^. Importantly, Smac mimetics also cooperated with Drozitumab in vivo in an orthotopic, intracranial mouse model to suppress glioblastoma growth^34^. On the molecular level, Smac mimetics and Drozitumab have been shown to act together to stimulate the assembly of a cytosolic complex containing RIP1, FADD, and caspase-8, which in turn resulted in caspase-8 and 3 activation^34^. Knockdown of RIP1 protein conferred protection from Smac mimetic/Drozitumab-mediated cell death, while RIP1 kinase activity or an autocrine/paracrine TNFα/TNFR1 signaling loop were found to be dispensable^34^. These findings point to a scaffold function of RIP1 protein in this model of cell death. Also, a small-molecule Smac mimetic of Smac binding to XIAP, cIAP1 and cIAP2 has been described to synergize with TNFα or TRAIL to induce caspase activation and apoptosis in human cancer cells including glioblastoma cells^35^. Furthermore, Smac peptides encompassing the N-terminal four amino acids that are highly conserved throughout evolution and critical for the interaction of Smac and IAP proteins have been shown to cooperate with recombinant TRAIL protein to induce apoptosis in glioblastoma cells in vitro and also in an orthotopic, intracranial glioblastoma model^31^.

Moreover, synergistic induction of apoptosis in glioblastoma cells has been described for the combination of TRAIL together with pharmacological inhibitors of PI3K/Akt/mTOR signaling^36^. Accordingly, PI3K inhibition by LY294002 broadly sensitized wild-type and mutant PTEN glioblastoma cells to death receptor-induced apoptosis following treatment with TRAIL or agonistic anti-CD95 antibodies as well as to chemotherapy-induced apoptosis in a highly synergistic manner^36^. Analysis of apoptosis signaling pathways revealed that LY294002 cooperated with TRAIL to cause drop of MMP, caspase activation, and caspase-dependent apoptosis^36^. Similarly, primary cultured glioblastoma cells generated from primary tumor samples were sensitized towards TRAIL by PI3K inhibition^36^.

Also, the combination of TRAIL and the proteasome inhibitor Bortezomib turned out to be a promising approach for inducing cell death in glioblastoma cells, including glioblastoma stem cells^37^. TRAIL and Bortezomib not only induced cell death in a highly synergistic fashion, but also suppressed long-term clonogenic growth of glioblastoma cells^37^. Mechanistically, TRAIL and Bortezomib acted together to increase accumulation of tBID, which resulted in enhanced activation of BAX/BAK and loss of MMP^37^. Interestingly, the stability of TRAIL-derived tBID has been found to markedly increase when the proteasome was concomitantly inhibited^37^. Knockdown experiments showing that BID silencing also significantly decreases Bortezomib- and TRAIL-mediated cell death confirmed the functional relevance of tBID in this model of cell death^37^. The potential clinical relevance was underlined by data showing that TRAIL and Bortezomib cooperated to trigger apoptosis in primary cultured glioblastoma cells and in patient-derived glioblastoma stem cells and to suppress tumor growth in an in vivo glioblastoma model^37^.

Moreover, pretreatment with distinct histone deacetylase inhibitors (HDACi) including MS275, suberoylanilide hydroxamic acid and valproic acid has been reported to significantly enhance TRAIL-induced apoptosis^38^. This synergy was shown to be mediated at least in part by c-myc-mediated downregulation of the antiapoptotic protein cFLIP^38^. MS275-mediated downregulation of cFLIP was preceded by upregulation of c-myc in the nucleus and occurred at the mRNA level, independently of proteasome- or caspase-mediated degradation^38^. This MS275-stimulated increase in c-myc resulted in enhanced binding of c-myc to the cFLIP promoter and suppression of cFLIP promoter activity^38^. Silencing of c-myc partially prevented cFLIP_L_ from MS275-inferred downregulation and significantly reduced TRAIL/MS275-induced apoptosis^38^.

One of the key advantages of proapoptotic receptor agonists targeting TRAIL R1 and R2 is their selective ability to kill malignant versus healthy cells^32^. Indeed, early clinical trial results confirmed that these agents are in principle well-tolerated^3^. However, they did not meet the expectation, as they also showed a rather limited therapeutic benefit^39^. This indicates that some shortcomings of initials trials need to be considered for the design of future, more effective treatment protocols.

## Targeting BCL-2 family proteins

**Fig. 3:**
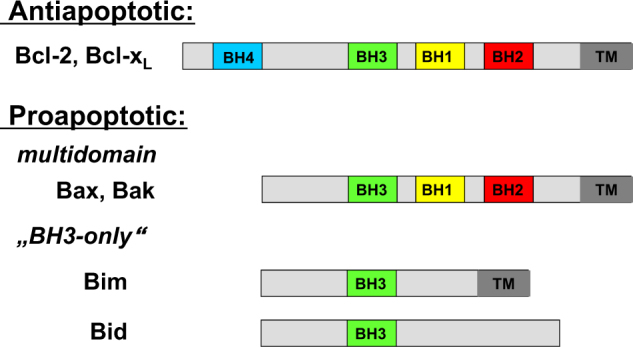
BCL-2 family of proteins

In the mitochondrial pathway of apoptosis, mitochondrial outer membrane permeabilization, (MOMP) leads to the release of mitochondrial intermembrane space proteins such as cytochrome c and Smac into the cytosol, where they promote caspase activation and caspase-dependent apoptosis^4^. MOMP is a tightly controlled event for example by proteins of the BCL-2 family. The BCL-2 family comprises on the one side antiapoptotic proteins (e.g., BCL-2, BCL-x_L_, and MCL-1) and on the other side proapoptotic members such as the multidomain proteins BAX and BAK and BCL-2-homology domain-3 (BH3)-only proteins such as BIM and NOXA that contain one single BH3 domain (Fig. 3)^40^. All members of the sub-group of BH3-only proteins share a nine amino acid BH3-domain, but otherwise possess very little structural homology^40^.

Activation of BAX and BAK is considered to contribute to MOMP as oligomerization upon their activation leads to formation of a channel in mitochondrial membranes large enough for the release of proteins from the mitochondrial intermembrane space^40^. In most cell types, BAX is located in the cytoplasm and, upon induction of apoptosis, translocates to mitochondrial membranes, while BAK is usually already associated with mitochondrial membranes even when non active^40^. Activation of BAX or BAK is opposed by their direct interaction with antiapoptotic BCL-2 family proteins such as BCL-2, BCL-x_L_, and MCL-1^40^. Antiapoptotic BCL-2 proteins have been reported to be expressed at high levels in malignant gliomas^41^. In apoptotic cells, transcriptional induction or posttranslational modification of BH3-only proteins can initiate activation of BAX and BAK.

Two competing models exist for explaining the activation of BAK and BAX by BH3-only proteins^42^. According to the direct activation model, activators among the BH3-only proteins (i.e., BID, BIM) directly bind to and activate BAX and BAK. In the indirect activation model, sensitizers among the BH3-only proteins (i.e., NOXA, PUMA, HRK, BMF) bind to pro-survival BCL-2 family members, which in turn displace them from BAX and BAK and thus, allows BAX and BAK activation and oligomerization and initiates MOMP.

High expression levels of BCL-x_L_ have been reported to be correlated with poor progression and survival of glioblastoma patients and BCL-x_L_ has been proposed as a marker of glioblastoma chemoresistance^43^. Targeting BCL-2 family proteins represents another approach for targeting the apoptosis pathways in glioblastoma.

BH3 mimetics represent a class of small-molecule inhibitors that mimic the activity of proapoptotic BH3-only proteins^44^. They target the BH3-binding domain of pro-survival BCL-2 family members by binding to a hydrophobic groove of antiapoptotic BCL-2 family members^44^. Several BH3 mimetics with distinct inhibitory profiles have been developed over the last decades. For example, ABT-199 selectively inhibits BCL-2, while ABT-263 antagonizes both BCL-2 and BCL-x_L_. The BCL-2/BCL-x_L_ inhibitor ABT-737 has been described to induce apoptosis in glioblastoma cells both in vitro and in vivo^45^. Downregulation of MCL-1 further enhanced the sensitivity of glioblastoma cells to ABT-737-triggered apoptosis^45^. Also, ABT-737 has been shown to increase the responsiveness of glioblastoma cells to chemotherapeutic agents and to the death receptor ligand TRAIL^45^.

Moreover, ABT-737 has been reported to cooperate with TRAIL to induce apoptosis in several glioblastoma cell lines, primary cultured glioblastoma cells derived from tumor material as well as in an in vivo glioblastoma model^46^. This synergy involved the cooperation of both agents to stimulate accumulation of the truncated form of BID (i.e., tBID) at mitochondrial membranes, BAX activation, drop of mitochondrial membrane potential, cytochrome c release and caspase-dependent apoptosis^46^. A mitochondrial feedback loop driven by caspase-3 also contributed to BID processing^46^.

In addition, ABT-737 has been found to synergistically induce apoptosis in combination with ionizing radiation in glioblastoma cells in a p53-dependent manner^47^. While wild-type p53 function has been found to inhibit the efficacy of combined treatment with ABT-737 and ionizing radiation, mutant p53 counteracted the proapoptotic activity of ABT-737 by maintaining MCL-1 expression levels^47^. Furthermore, the PI3K/Akt inhibitor NVP-BKM120 has been shown to improve the anticancer activities of ABT-737 in established and primary cultured glioblastoma cells^48^.

Pharmacological or genetic inhibition of the HSF1/HSP70/BAG3 pathway using the selective HSF1 inhibitor KRIBB11, the HSP70/BAG3 interaction inhibitor YM-1 or BAG3 silencing has been reported to significantly enhance cell death upon treatment with the pan-BCL-2 inhibitor AT-101^49^. Currently, some BH3 mimetics such as ABT-199 are being evaluated in clinical trials, in particular in hematological malignancies^44,50^.

## Systems medicine approach for targeting cell death in glioblastoma

Recently, a systems medicine approach has been developed to investigate the ability of glioblastoma cells to execute apoptosis and has been used to predict treatment responses of glioblastoma patients^51^. This approach has led to the identification of executioner caspase activation as a predictor of progression-free survival in glioblastoma patients^51^. Accordingly, a systems biology-based mathematical model, i.e., APOPTO-CELL, that takes into account expression levels of a panel of key proapoptotic proteins (e.g., Smac and XIAP) has been generated to predict cellular susceptibility to undergo caspase activation^51^. APOPTO-CELL has been found to accurately discriminate between glioblastoma cells that undergo apoptosis upon TMZ treatment or, alternatively, fail to respond^51^. Of note, APOPTO-CELL was able to stratify glioblastoma patients with respect to progression-free survival times^51^. Thus, a systems medicine approach to predict the capability of cancer cells to execute apoptosis may open new perspectives to predict treatment responses.

## Conclusions

Reactivation of programmed cell death represents a promising therapeutic strategy for human cancers including glioblastoma. One prime example of how cell death pathways can be targeted for therapeutic purposes is the development of the BCL-2 inhibitor ABT-199 that has recently received breakthrough status by the Federal Drug Administration for the treatment in chronic lymphocytic leukemia and acute myeloid leukemia (Box 1). However, no such highlight has so far been achieved in glioblastoma as compared to other cancer entities, although there are several distinct approaches to interfere with cell death signaling pathways that are transferable into the clinical setting. One of the remaining challenges resides on the identification of suitable predictive biomarkers for the selection of patients that likely will benefit most from a given experimental protocol. Also, drug combination strategies will have to be taken into consideration to maximize the antitumor activity of cell death-inducing agents, as monotherapy with these agents will likely not be sufficient. For example, combining cell death-targeting drugs together with immunotherapy is particularly attractive. For example, Smac mimetics have recently been shown to synergize with immune checkpoint inhibitors to suppress tumor growth in mouse models of glioblastoma^23^. Such strategies may pave the avenue for new options for the treatment of patients suffering from glioblastoma.
